# Consultation meeting on the development of therapeutic vaccines for post kala azar dermal leishmaniasis

**DOI:** 10.1186/1475-9292-6-7

**Published:** 2007-08-17

**Authors:** Hashim Ghalib, Farrokh Modabber

**Affiliations:** 1 UNICEF/UNDP/World Bank/WHO Special Programme for Research and Training in Tropical Diseases (TDR), Geneva, Switzerland; 2 Centre for Research and Training in Skin Diseases and Leprosy, Tehran University of Medical Sciences, Tehran, Iran

## Abstract

**Background:**

Post kala azar dermal leishmaniasis (PKDL) is a disease that appears after treatment of visceral leishmaniasis (VL). The highest incidence of PKDL in the world is in Sudan. Many patients heal spontaneously within 6 months but those who don't are difficult to treat, often requiring months of daily injections. These patients harbour parasite in their skin and are believed to be a source of infection and possibly epidemics. Present treatment modalities of PKDL are inadequate and impractical due to cost, duration of treatment required and side effects. New approach for treatment of PKDL is required. A joint meeting of the UNICEF/UNDP/World Bank/WHO Special Programme for research and training in Tropical Disease (TDR) and the Infectious Disease Research Institute (IDRI) Seattle, USA was held to review the progress of therapeutic vaccines and plan the development of treatment modalities for PKDL.

**Methods:**

The history of leishmaniasis vaccine development for prophylaxis and therapy was reviewed. Other than previous infection – simulated by inoculation of live Leishmania as a vaccine (leishmanization), none of the preparations of killed parasite with or without adjuvants have shown significant prophylactic efficacy. Killed L. major absorbed with alum and mixed with BCG remains to be tested as a prophylactic vaccine.

**Results:**

Killed parasite preparations i.e. L. mexicana mixed with BCG and L. amazonensis (combined with low dose of antimonial), have shown efficacy in immunotherapy and immuno-chemotherapy, respectively. In addition combined full antimonial plus alum-absorbed autoclaved L. major vaccine has been shown to significantly improve therapy of refractory PKDL patients. These are all crude preparations of parasites and are difficult to define and standardize. However, there is now a new, second generation vaccine, Leish-111f + MPL-SE, composed of a recombinant protein comprising three leishmanial antigens and a defined adjuvant in clinical development.

**Conclusion and recommendations:**

Immuno-chemotherapy has the potential of becoming a practical and affordable treatment modality for PKDL and other forms of leishmaniasis. The encouraging results with alum-autoclaved L. major + antimonial should be pursued. However, before further trials, availability of the vaccine and its production under Good Manufacturing Product, hence quality control must be assured. Following satisfactory safety profile of Leish-111f+MPL-SE, clinical trials using this vaccine initially with antimonials should be initiated. Similarly immunotherapy of VL should be considered with the view to controlling development of PKDL. Some immunological studies are required prior to initiation of immunotherapy in VL patients.

## Background

Dr R. Ridley, Director of TDR, opened the meeting and pointed out the importance of immunological interventions for treatment of diseases. The therapeutic potential of vaccines is easier to assess than the prophylactic efficacy (quicker readout, smaller sample size, etc.) and may be used as a screening mechanism to select candidate vaccines for prophylaxis, in this case against different forms of leishmaniasis. Dr Ridley noted the initiative of the ministries of health of India, Nepal and Bangladesh to eliminate leishmaniasis (as a public health problem) by 2015. Clearly several tools will need to be added to the new drugs and diagnostic tests recently developed and now being evaluated. In addition, better treatment modalities for VL and a practical treatment for PKDL would be highly valuable.

Dr J. Lazdins, Coordinator PDE, indicated that use of immuno-chemotherapy for increasing the efficacy of medication and preventing an increase in drug-resistant organisms is a logical approach. He was particularly interested in the recommendations from this meeting in connection with consideration of combined therapy for other TDR target diseases.

Dr Ghalib, Coordinator of Leishmaniasis Research (TDR), presented the strategic research emphases of TDR for 2006–2007 and indicated that the shaping factors would be publication of the Tri-Tryp genome and setting up of the control initiative in South Asia. The first is a 'research pull' to provide a wealth of information useful for development of new tools. The second is a 'control pull' which demands new tools and comprehensive efforts toward controlling VL in South Asia. These are opportunities to be seized.

Dr Ghalib noted that new effective drugs have appeared (Miltefosine, Aminosidine, Ambisome) hoping that they will become more affordable. However, he warned that these are being used as single drug therapy. Use of combined therapy including immuno-chemotherapy is urgently needed to safeguard the new drugs against resistance.

Dr Ghalib discussed the new tools on the horizon, including a new erythrocyte binding assay based on chemical changes to the surface of red blood cells during active VL disease, which was developed by Dr. Mandal at the Indian Institute of Chemical Biology and has been adapted into a dipstick format. Evaluation of the rK39 dipstick test, direct agglutination test (DAT), and latex agglutination urine test has now been completed in Ethiopia, Kenya and Sudan. The results from these sites are being analysed. Dr Ghalib indicated that the purpose of the present consultative meeting was to review progress and available tools, and to recommend how to proceed towards developing practical and affordable immunotherapeutic modalities for treatment as well as prevention of PKDL.

Dr. Ghalib introduced to the participants the various forms of leishmaniasis and their public health significance in the world. The overall prevalence is estimated by WHO to be 12 million with 350 million at risk. Ninety five per cent of VL cases occur in five countries (India, Bangladesh, Nepal, Sudan, and Brazil) and 90% of CL cases occur in eight countries (Afghanistan, Algeria, Brazil, Iran, Iraq, Peru, Saudi Arabia, Syria). There are about 20 *Leishmania *species that cause disease in humans and about 30 species of sandfly vectors. Based on the complexity of the different epidemiological settings and the superimposed problem of co-infection with HIV in some parts of the world, it is no surprise that the conventional tools have failed to control leishmaniasis. New tools and better use of available tools are needed. Recent endeavours have been undertaken and new data accumulated on the use of vaccines both for prevention and therapeutic use. Prophylactic vaccines for leishmaniasis are being planned under the Initiative for Vaccines Research (IVR) at WHO, while immunotherapeutic vaccines for PKDL, VL, and VL-HIV are the topics of interest to TDR. At present the vaccines shown to be effective in protecting animals are: a whole killed parasite +/- adjuvants (first generation vaccine); a purified or recombinant vaccine (protein or DNA) plus adjuvant; a live attenuated *Leishmania*; and a live virulent *L. major*. Below we will consider those vaccines that have entered clinical development for human use. Hence experts with experience in human leishmaniasis vaccines were convened for this meeting in order to review the available data and recommend avenues to be explored in developing a safe, efficacious and affordable vaccine, with a focus on immunotherapy of VL and PKDL.

## Presentations

Please see Additional file 1 for list and addresses of all contributors.

Professor Zijlstra, Chairperson of the meeting, described the epidemiology of CL and VL [1] and the natural history of VL with ensuing PKDL in Sudan [2]. Whereas PKDL develops in 50%–60% of VL cases in Sudan, the incidence of reported PKDL cases remains at about 0.8%–8% in India.

Inadequate treatment may be a factor in the development of PKDL; however, even with complete treatment to cure, PKDL may develop [3]. Other factors such as genetics, the organism, nutrition, etc., may be important. Presentation of PKDL is diverse, may develop in a few cases without previous obvious VL, and can involve other post kala azar manifestations in oral, ocular and nasal tissues. In children, severity is inversely related to age; the majority of patients with grade 3 severity are generally children less than 8 years old. Response to treatment does not seem to be associated with severity, and the majority of cases self heal. About 40%–45% of cases in Sudan do not cure spontaneously within 6 months and about 20% still have PKDL after a year [3]. PKDL patients require several months of treatment with daily injections of antimony. This is not affordable or practical for patients. As it is well known to the local populations that PKDL, unlike VL, is not life-threatening, people take it as a good sign, believing that the deadly disease has surfaced and will not be able to kill. Treatment is therefore not sought. The role of PKDL as a reservoir has not been directly proven in Sudan (but is well documented in India); however PKDL patients do harbour parasites in their skin [4]. Therefore treatment of PKDL should be considered as an important public health measure for controlling VL.

Professor EAG Khalil presented immunopathology and chemotherapy of VL and PKDL in Sudan. He described the wide range of disease manifestations from localized skin lesion (leishmanioma) to severe cutaneous disfiguration by PKDL, even uveitis and blindness, psychological and nervous system involvement. He indicated that in severe VL no organ is spared, and discussed VL-associated pathological manifestations in different organs including lymphadenopathy, splenomegaly, hepatomegaly, immune-complex associated glomerulonephritis, pneumonia and tuberculosis. He contrasted the polarized Th1–Th2 responses of the mouse with those in human leishmaniasis, in which the two responses overlap, and indicated that genetic factors, nutrition and the amount of antigen may determine the type of response. Both Th1 and Th2 antigen specific cells are present in cured VL patients. Nevertheless, during active disease, interleukin-10 (IL-10) levels increase in lymph node cells and peripheral blood mononuclear cells (PBMC) [5]. Addition of IL-10 inhibits proliferation of PBMC from cured patients to leishmanial antigens. The balance between Th-1/Th-2 responses in progression of disease (Th2 predominance) and cure (Th1 predominance) was stressed.

Professor S. Sundar reviewed different treatment modalities of VL in India. The efficacy of first-line drugs (pentavalent antimonials) had dropped from 84% in 1988 to less than 40% in 2002 [6]. A similar trend is being seen in Nepal. The rate of drug-related deaths, primarily due to cardiotoxicity, is 3%–5%. Pentamidine, used as second-line drug for years, is not being used due to toxicity. Amphotericin B is used only in hospitals due to its toxicity, and its use is limited because most patients cannot afford it. Lipid-associated amphotericin B has limited toxicity due to lower doses being efficacious, and because there is less circulating drug as the lipid complex is taken up preferentially by the reticuloendothelial system [7,8]. Ambisome, the best drug available, which can cure about 90% of VL cases with a single injection, is far too expensive [7].

Recent work on the new (the first) oral drug for VL, Miltefosine, was presented. Using 100 mg/day for patients over 25 kg body weight (or 2.5 mg/kg for children) for four weeks, the initial cure rate was very high (98%) with moderate and acceptable side effects. Hospitalization is not required, which reduces the cost of treatment considerably. However, with home-based treatment lasting for four weeks, compliance is likely to be low when a patient feels well after 1–2 weeks of treatment unless a DOT-like treatment system is implemented. In a recent home-based trial on 367 patients, a relapse rate of 6.7% was observed, which might be the result of lower compliance compared to the original hospital-based trial. The present cost is also a concern. In addition, miltefosine is teratogenic in animals, hence should not be given to women of childbearing age without adequate contraception.

Aminosidine (paromomycin) is a more affordable drug with few side effects. In a phase-III comparative trial with Amphotericin B, 95% cure was achieved with 21 daily injections of Aminosidine (15 mg/kg/day) assessed after 6 months. This represented comparable efficacy but with far fewer side effects than Amphotericin B. Aminosidine is less costly and would be a good replacement for antimonials.

Sitamaquine (WR6026), an oral drug originally discovered by the Walter Reed Army Institute of Research, was initially tested in a limited number of patients in Kenya. A phase-II dose finding trial indicated 80%–100% cure rates. Further studies are planned.

Prof. Sundar summarized the therapeutic options for India as shown below and emphasized that, to prevent development/escalation of drug failure, it is recommended that combination therapy (drugs or drug + vaccine) be developed.

### Summary of therapeutic options

• Use antimony only in responsive regions.

• Amphotericin B is the most important drug available and gives >95% cure rate. As Ambisome is extremely expensive, Amphotericin B or a new formulation of stable lipid-Amphotericin B at affordable price is needed.

• Miltefosine (oral):

- effective and safe

- price should be reduced to affordable levels, even in the private sector

- is teratogenic in animals, with a long half life (rapid emergence of resistance)

- is costly and not affordable (Rs 6600.00, about US$ 1480)

- is there compliance with domiciliary care (DOT)?

• Paromomycin

- are quite promising, and an appropriate substitute for antimony.

• A combination of drugs (to achieve a short treatment regimen) is required.

## First generation vaccines

Professor F. Modabber reviewed the global experience with first generation vaccines (whole parasites with or without BCG as adjuvant) used for prophylaxis or combined with antimonials for immuno-chemotherapy [9-11]. Trials in Brazil, Colombia, Ecuador, and Iran were presented. The trials conducted in Sudan were presented by Prof Khalil and Dr Musa and those in Venezuela by Dr Ulrich (see below).

For prophylaxis, live virulent *L. major *(leishmanization) is the best vaccine against human cutaneous leishmaniasis. It has been used for centuries, much like cow pox, by inoculating material from exudates of an active lesion into a covered part of the body. In more recent years in Iran, Israel and Uzbekistan, leishmanization has been performed by inoculating cultured *L. major*. Leishmanization cannot be used on a large scale or in HIV endemic areas. Since *Leishmania *undergo biological changes in culture, lose their infectivity when sub-cultured, and cannot be lyophilized, they must be kept frozen in liquid nitrogen, and hence their delivery is impractical. However, if inoculates are standardized and used under special conditions in non-HIV infected areas, then leishmanization could be used as a live challenge system in order to assess the efficacy of candidate vaccines [12].

Various killed parasites have been tested for decades with or without different adjuvants. None of the killed parasites has been shown to be sufficiently efficacious as a prophylactic vaccine to be used in control programmes [13]. The parasites tested in this way include *L. amazonensis *(tested in Colombia by Velez et al.) [14], and *L. mexicana *+ BCG (see below). Autoclaved *L. major *(ALM) + BCG and its alum-absorbed preparation (alum-ALM) have been tested in monkeys using BCG [15]or IL-12 as adjuvants [16], and in clinical trials against CL in Iran [17,18] and against VL in Sudan [19] (see below).

### First generation vaccines for prophylaxis

#### Killed *L. major *+ BCG with or without alum in Iran

Initially different methods of killing promastigotes of *L. major *were tried; finally autoclaving was used much like that reported by Convit et al. in Venezuela. The Razi Serum and Vaccine Institute, Iran, produced the vaccine, and the Pasteur Institute, Tehran, provided BCG (used for TB vaccination in the country). Following several safety, dose escalation and immunogenicity trials in non-endemic foci of Iran, one injection of 1 mg ALM mixed with 1/10^th ^of the BCG dose normally used as TB vaccine was chosen for trials against anthroponotic and zoonotic CL in Iran [17,18]. A series of double-blind randomized BCG-controlled trials with 1–3 injections of the vaccine were conducted. No significant overall difference was seen between the vaccine group and the control (BCG alone) group. However, those who had become leishmanin skin test (LST) positive were found to have significant protection. Subsequently, ALM was absorbed to alum as Kenney and colleagues at NIH had shown this preparation to be far more immunogenic and to produce a strong and long-lasting Th1 response and protection in mice and monkeys [16]. The new formulation went through safety, dose-escalation, immunogenicity and protection studies against VL in langur monkeys in India [15]. Results were highly encouraging and indicated clinical development should continue. Following safety studies in Iran, this preparation was tested in Sudan (see below). A trial was also conducted in Iran using one injection of alum-ALM + BCG in volunteers who then received leishmanization as challenge infection. This trial did not produce conclusive results due to low virulence of the live *Leishmania *stabilates used for leishmanization and low virulence of the BCG used. Whereas the stabilates had produced 85%–100% lesions in two previous trials a few years before, the take rate in this trial was only about 46%. The trial is due to be repeated with new standardized *Leishmania *stabilates.

#### ALM + BCG (without alum) in Sudan

Professor EAG Khalil presented his team's experience with first generation vaccines. Two preparations – autoclaved *L. major *(ALM) + BCG, and alum-ALM + BCG – were tested as candidates for prophylactic vaccines against VL [19]. A series of dose escalation, safety and immunogenicity studies were conducted in Iran, where the vaccines were produced (Razi Institute). The results were satisfactory, and safety and immunogenicity studies were then conducted using one and two injections of 1 mg + 1/10^th ^of normal BCG dose used for TB vaccination in healthy adult volunteers before embarking on a field efficacy trial in a hyperendemic area (Gedaref state) in Sudan. The logic behind using an *L. major *vaccine against VL is based on the observation that people who are leishmanin skin test (LST) positive and come from CL hyperendemic zones have immunity against VL (suggested from the results of a prospective epidemiological study) [1]. Secondly, LST conversion induced by the vaccine seems to be associated with resistance to leishmaniasis (data from Brazil and Iran), and the killed vaccine induces LST reactivity. The results of a double-blind, BCG-controlled study involving 2306 volunteers, randomly allocated to receive two doses of ALM + BCG or BCG alone with two years of follow-up, can be summarized [19] as follows:

• Side effects were minimal.

• Cumulative incidence rates of visceral leishmaniasis at two years were not significantly different between the two study groups (P = 0.6).

• However, the incidence of VL was significantly lower in those individuals whose LST became positive (induration ≥ 5 mm) following vaccination compared to non-responders (7.2%, vs. 12.7%, respectively; P = 0.0003).

These results were encouraging but the vaccine was not sufficiently immunogenic to be used in control programmes.

Subsequently, it was shown that absorption of ALM to alum greatly enhances its immunogenicity and protective potential against CL in mice and VL in langur monkeys.

#### Alum-ALM + BCG in Sudan

Two dose-escalating safety and immunogenicity studies were conducted by Prof Khalil and the Leishmaniasis Research Group, Sudan. There were 24 and 44 healthy volunteers in the two trials respectively, and results of the two studies were essentially the same [20,21]. The vaccine was safe and side effects were confined to the site of injection. A single injection of the vaccine at all doses was safe and immunogenic as measured by LST conversion and IFN-γ, but there was no significant IL-10 or IL-5 production. The vaccine induces a strong Th1 response with no significant antibody production in almost 100% of volunteers by day 60. A single injection produces a stronger delayed-type hypersensitivity (DTH) response than even multiple injections of ALM + BCG (no alum). The alum-ALM preparation without BCG did not induce skin test conversion indicating that a T-cell adjuvant is required for DTH response. Based on the results using different doses in the two trials, 100 μg alum-ALM + BCG was selected as the most suitable dose to use in efficacy trials. An extended safety and immunogenicity study was conducted under field conditions; the trial included some children who are at high risk of VL. The results indicated that the vaccine was safe and well tolerated under field conditions. The LST conversion rates were 56%, 50%, 25% and 31% at 6, 12, 18 and 24 months post vaccination respectively. Conversion rates for the BCG control group were 4%, 12%, 3% and 13% at the same intervals respectively. There was no significant antibody rise at follow-up visits (p < 0.05). These are very encouraging results. Further studies with multiple injections would be of interest. This vaccine was used for immuno-chemotherapy of PKDL patients (see below). Prof Khalil summarized his future plans for this vaccine as follows:

• Conduct phase I/II studies of multiple doses of alum/ALM + BCG.

• Conduct a prophylactic efficacy trial with alum/ALM + BCG against VL.

• Conduct an efficacy trial for immuno-chemotherapy of PKDL (see below).

The experience with killed *L. major *vaccine can be summarized as follows:

• ALM + BCG is safe, affordable, and induces 8%–17% LST conversion in populations of endemic foci and 30%–37% in non-endemic areas. It produces a weak Th1 response but no significant Th2 response.

• Overall, the immunogenicity and protection induced against CL (zoonotic or anthroponotic) or VL is not sufficient to use the vaccine as a prophylactic in control programmes. However, LST converted recipients have a significantly lower incidence of disease.

• No further clinical trials should be considered for this preparation.

• Addition of alum to ALM + BCG however (alum-ALM + BCG) makes it highly immunogenic. Alum-ALM + BCG induces long-lasting LST conversion in almost 100% and 56% of non-endemic and endemic populations respectively.

• A trial on the prophylactic efficacy of the alum-ALM + BCG compared with leishmanization was inconclusive and should be repeated with new stabilates of live *L. major*.

• Alum-ALM + BCG (multiple injections) should be studied as a prophylactic vaccine against VL in Sudan.

• Addition of alum-ALM + BCG to standard antimonial therapy for patients with persistent PKDL significantly enhances cure (see below).

• Alum-ALM with BCG or other adjuvants (CpG) has induced strong and long immunity and protection in mice and primates and should be considered as a strong candidate for human prophylactic studies.

### First generation vaccines for treatment

Although the first generation vaccines have not been efficient for prophylaxis, three formulations have shown efficacy alone or when combined with chemotherapy for the treatment of leishmaniasis. These are:

#### Killed *L. amazonensis *in Brazil

**Figure 1:**
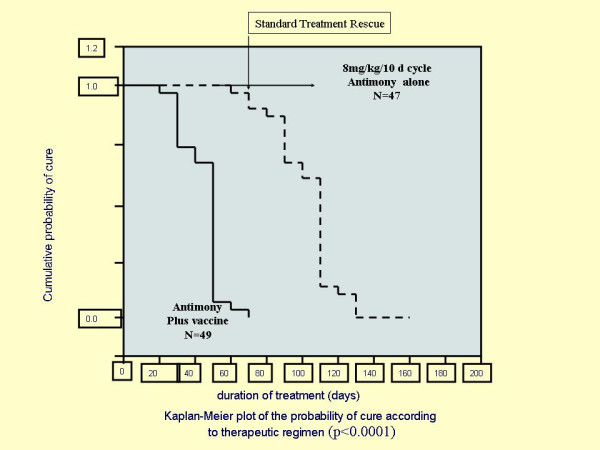
Patients received daily injections of low dose antimonial + killed vaccine for 10 days followed by 10 day rest (one cycle). After 4 cycles combined treatment cured all patients vs. 8% in low chemo alone. Combined treatment significantly reduced drug dose, cost and time to cure. The vaccine was registered in Brazil. Adopted from Machado-Pinto J. *et al. Int. J. Dermatol*. ***41**:73-8, 2002*. with permission from Oxon Blackwell Pub. .

Administration of vaccine (Mayrink's vaccine produced by Biobras) together with daily injection of a reduced dose of antimonial cured 100% of CL cases in Brazil compared to only 8% given low-dose antimonial only during the same period (80 days). The results of this trial by J. Machado-Pinto et al. (Fig. 1) were used to register the vaccine as an adjunct to low-dose chemotherapy with antimony [22].

#### Killed *L. mexicana *+ BCG in Venezuela

Dr M. Ulrich presented studies on immunotherapy with autoclaved *L. mexicana *+ BCG which were conducted in Venezuela. Following the initial trials in the 1990s, more than 30000 patients have received immunotherapy in Venezuela. Dr Ulrich outlined the findings from a trial involving 5341 patients. The dose of BCG was adjusted according to purified protein derivative (PPD) skin reaction, 0.1 mg for PPD-negative and 0.01 mg for PPD-positive patients [9]. Patients were injected intradermally in two sites in the deltoid areas with 0.5 ml of a suspension containing 6.4 × 10^8 ^autoclaved promastigotes of *L. mexicana *MHOM/VE/84/MEL and freshly resuspended BCG (Statens Seruminstitut, Denmark). In later practice, patients were given one or two additional injections at the discretion of the treating physician. Clinical cure ranged from 91% to 98% in the different trial sites. Booster injections significantly shortened the time to cure: three injections cured 98% of patients within 24 weeks, while only 18% and 38% were cured at this time with a single or double injection respectively. More recently, pasteurized rather than autoclaved *L. braziliensis *plus BCG was shown to significantly reduce the time to cure (in 277 patients) as compared to autoclaved vaccine (56 patients) [10]. Some preliminary data with 2-D electrophoresis indicate that proteins are better preserved in pasteurized preparations.

In conclusion:

• Immunotherapy with killed promastigotes plus BCG is highly effective in treatment of localized cutaneous leishmaniasis (LCL) in Venezuela.

• Preliminary studies suggest that pasteurized promastigotes produce more rapid healing (perhaps due to better conservation of antigens).

#### Killed Leishmania major plus alum plus BCG for treatment of PKDL

Dr AM Musa described two observations that led to the use of the alum-ALM + BCG vaccine as an immunological stimulus for treatment of patients with persistent PKDL:

• LST positive PKDL patients have a favourable prognosis.

• Alum-ALM + BCG vaccine induces a strong LST reaction.

A series of dose escalation safety studies with single and multiple injections were conducted using alum-ALM + BCG in volunteer patients with persistent PKDL who were under antimony treatment. Together with the data collected on healthy volunteers described by Prof. Khalil, four doses of 100 μg alum-ALM + BCG were selected for a proof-of-principle study to show if the addition of vaccine could reduce the dose of antimonials required for patients with persistent PKDL (3–4 months of daily injections with antimonials). This was a hospital-based, blind, randomized, controlled trial. Thirty patients with persistent PKDL were brought from the endemic area to the hospital in Khartoum and randomly assigned to receive either 40 injections of 20 mg/kg/day (chemotherapy) or chemotherapy + four weekly injections of 100 μg alum-ALM + BCG (combination). Patients were evaluated at the end of therapy (day 40) to determine the course for further treatment, either continued chemotherapy, or no treatment but observation or rescue treatment with Ambisome in failed cases. Patients were again evaluated on day 60, after which they were discharged.

**Figure 2:**
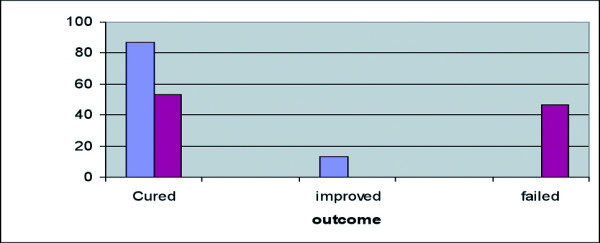
Immunochemotherapy of PKDL. Patients received either 40 daily injections of 20 mg/kg/day Sodium Stibogluconate (SSG) alone, or SSG plus 4 weekly injections of alum-ALM+BCG. Cured = complete disappearance of lesions (not including discoloration or scar).

Combination therapy cured 86.6% of patients at the end of treatment (day 40), while the remaining 13.4% were improved and were observed until day 60 but required no more treatment. Chemotherapy alone cured only 53.3% at the end of treatment, while 46.7% were not cured at this time and received continued chemotherapy or rescue treatment. There were no failures in the group with combined therapy (Fig 2).

A general trend was seen in conversion to LST-positive reaction or increase in LST with progression to cure. A mixed Th-1 and Th-2 immune response was observed in the cytokine patterns, with a trend towards increased IFN-γ and decreased IL-10. These immunological data must be considered as an indication rather than proof since the sample size was small and patient-to-patient variations large. However, clinical cure rates were significantly different in the two groups.

## Second generation vaccines

Dr S. Reed, President of IDRI, reviewed the background and purpose of his institute and its work on diseases of disadvantaged populations in developing countries. IDRI was founded in 1993 and focuses on vaccines and diagnostics. For leishmaniasis vaccine, IDRI collaborated with a biotech company (Corixa, now GSK) which had T-cell adjuvants needed for vaccine development. He explained that an ideal leishmaniasis vaccine would be one that produces a Th1 type response to selected 'protective' antigens and long-term immunity against different species of *Leishmania*, and one that is safe, therapeutic and cost effective. To this end, Reed and colleagues initiated antigen discovery and realized that not all antigens can protect in mouse model. From over a dozen antigens, IDRI selected three protein antigens [23]. Two of these (LSTI1, TSA) were selected by expression cloning with human or mouse sera [24-26]. They were also tested by reactivity of T-cells from recovered/cured leishmaniasis patients and finally validated in mouse protection studies [25,27,28]. The third antigen (LeIF) is not protective in mouse models but was selected on the basis of its unique characteristic of inducing IL-12 (and thereby IFN-γ) and IL-18 in mouse and human monocytes and fibroblasts [29-31]. LeIF is an internal adjuvant for Type 1 immune response, and should be useful for prophylaxis as well as therapeutic use. Methods for identification of the three selected antigens are shown below.

• LeIF – a ribosomal initiation factor – identified by serological screening with human sera from healthy, infected, DTH positive individuals.

• LmSTI1 – a temperature inducible protein – identified by serological screening with sera from BALB/c mice infected with *L. major *(abundant and immunogenic antigens).

• TSA – a thiol-specific antioxidant – identified by serological screening with sera from BALB/c mice immunized with protective leishmanial antigens.

**Figure 3:**
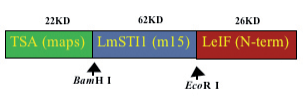
Schematic representation of Leish-111f construct containing tandemly linked genes of TSA, LmSTI1 and LeIF.

The genes of these three antigens were tandemly linked and expressed to produce a single protein with the three subunits [32]. The construct (Leish-111f, shown in Fig 3) has all the activities of the components and was produced by Corixa under good manufacturing practice (GMP) standards for human use.

After testing a dozen adjuvants in protection studies with Leish-111f in mouse models, monophosphoryl lipid A of *Salmonella minnesota *(MPL) was selected. It activates innate immunity through a TLR-4 receptor, enhances phagocytosis, up-regulates major histocompatibility complex (MHC) expression, and induces a cascade of cytokines [33]. MPL has been shown to be safe in aqueous solution and has been used in several human vaccines as SBAS2 formulation, including in malaria, Epstein-Barr virus, herpes simplex and hepatitis B vaccines. It has been given to over 33300 individuals including young adults, the elderly, and adolescents. In the IDRI Leish-111f vaccine, MPL is used as a stable emulsion in squalene (SE) to enhance its Th1 stimulation over Th2 (seen in aqueous form). Dr Reed reviewed the pre-clinical studies with individual antigens and the final vaccine, Leish-111f + MPL-SE. Based on data submitted, an investigational new drug (IND) approval was granted by the US Food and Drug Administration (FDA) to initiate human phase-1 clinical studies in the USA and subsequently in Latin America.

Professor R. Badaro presented data on the use of a mixture of four antigens discovered by IDRI together with GM-CSF (as adjuvant) on a compassionate basis for treatment of refractory cases of mucosal leishmaniasis (ML). This work was done at the Federal University of Bahia, Salvador, Brazil, in collaboration with IDRI and Corixa [34,35]. The four antigens were the three antigens later used to construct Leish-111f, plus 6H (LmHSP 83), which was chosen on the basis of its strong human T-cell stimulation. He first described cytokine patterns in CL patients before and after cure either by heat or antimonial treatment. He then reviewed data on the limited trial with Mayrink's vaccine (killed parasite) and mentioned the concerns about using material to which ML patients exhibit a strong DTH reaction. He then described a patient who had exhausted all treatment possibilities without success and was desperate to the verge of suicide. Following impressive success with this patient, ten more patients were treated with different numbers of injections of the vaccine (5 μg each of TSA, LmSTI1 and Lbhsp83, 10 μg LeIF, plus 50 μg GM-CSF) and/or received additional antimonial injections. The vaccine was safe and well tolerated. Except for expected side effects related to GM-CSF (flu-like symptoms) and local irritation at the site of vaccination, no adverse reaction was observed. All patients were cured and remained without a lesion for 5–8 years of follow-up. This was an open study, and treatment (vaccine and or chemotherapy) was given at the discretion of the treating physician.

**Figure 4:**
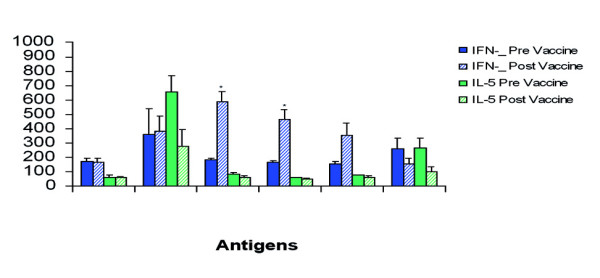
Cytokine responses of ML patients. Peripheral Blood Monocytes of patients were analysed for production of cytokines *in vitro *when stimulated with different antigens, before and after vaccination. Note the increase IFN-γ production to specific antigens (TSA, LmSTI1 and LeIF) after vaccination.

Some of the patients donated blood samples that were analyzed for cytokine responses to the four antigens plus soluble leishmanial antigens (LSA), It is of particular interest that there was enhancement of IFN-γ and inhibition of IL-5 to LmSTI1, TSA and LeIF (the three components of Leish-111f) after vaccination. There is also reduction of IL-5 to soluble *Leishmania *antigens (SLA) (whole parasite antigens) after immunization associated with cure, but there is no change in IFN-γ production (Fig. 4). The IFN-γ level produced by these antigens was already high prior to vaccination. This is in line with the concept that cure is accomplished, or at least accompanied by, a shift to a Th1 type response to leishmanial antigens, and that IFN-γ production to certain, but not all, antigens is important for immunity against leishmaniasis. Another interesting observation was the skin test conversion of most patients after immunization with the vaccine antigens (not LeIF). Enhanced DTH reaction to leishmanin post vaccination is not as obvious since most patients already had a strong (even allergic) reaction to leishmanin in contrast to their reaction to the recombinant antigens.

### Dose-escalating safety and immunogenicity trial in healthy volunteers in the US

Dr F Piazza presented the protocols for clinical trials of the Leish-111f + MPL-SE vaccine in the US. The first clinical trial was conducted in the State of Washington, USA, under an IND of the US FDA. The endpoints were the safety of three injections of 10 μg, 20 μg or 40 μg Leish-111f mixed with 25 μg MPL-SE in healthy adult volunteers of both sexes. This was a three cohort, sequential, dose-escalating, randomized, double-blind, controlled trial. Each dose cohort consisted of nine volunteers injected with full vaccine, three with adjuvant alone and three with saline, so that at the end of the trial there would be nine volunteers in each group. Following satisfactory safety results up to day 84 of the first cohort (10 μg dose), the next cohort (20 μg) would enter the trial. Similarly, the last cohort of 40 μg was entered when satisfactory safety outcomes were observed at day 84 of the second cohort. Follow up was both passive, by a diary kept by the volunteers, and active, by physical examination at pre-set intervals and collection of blood specimens. The results are summarized below:

• 41 (91%) of 45 subjects received all three injections.

• All subjects had at least one adverse event (AE), 80% of which were mild.

• The most common AEs were limited to the site of injection (SOI).

• SOI reactions were erythema, induration and pain.

• No subject experienced a severe AE during the study injection period.

• Four subjects (one in the 20 μg vaccine group, and three in the 40 μg vaccine group) experienced grade 3 AEs.

One volunteer in cohort 2 who had received adjuvant alone died of myocardial infarction. After reviewing the data (by safety monitor) it was decided that the death was not related to vaccination and the third cohort was injected with the higher dose. There was no serious AE in cohort 3. Overall, the vaccine is reactogenic but safe. Following analysis of the safety of the vaccine in healthy individuals, two safety and immunogenicity trials were initiated in patients with active CL or ML.

### Dose-escalating, safety and immunogenicity trial in patients with ML in Peru

Prof A. Llanos-Cuentas described the ongoing double-blind, three-cohort randomized, placebo-controlled, dose escalating, safety and immunogenicity trial of Leish-111f + MPL-SE in patients with ML in Peru. The cohorts and injection assignments are shown in Table 1. All patients received the standard treatment (Sb^v^) in addition to the injections indicated in Table 1. As part of safety, lesion evolution is being assessed to note any possible exacerbation of the disease. As this is a blinded trial, global results encompassing both arms were presented.

**Table 1:** Treatment Groups in Patients with Mucosal Leishmaniasis (Peru)

**Cohort**	**N**	**Treatment**	**Timing of Treatment**	
			
			**Study Injections**	**Pentavalent Antimony (Sb^5+^)**
# 1	12 4	5 μg Leish-111f in 25 μg MPL-SE + Sb^5+^ Placebo (saline) + Sb^5+^	Day 0, Day 28 and Day 56	SSC^1^
# 2*	12 4	10 μg Leish-111f in 25 μg MPL-SE + Sb^5+^ Placebo (saline) + Sb^5+^	Day 0, Day 28 and Day 56	SSC^1^
# 3**	12 4	20 μg Leish-111f in 25 μg MPL-SE + Sb^5+^ Placebo (saline) + Sb^5+^	Day 0, Day 28 and Day 56	SSC^1^

^1^SSC = Standard single cycle: 28 days of pentavalent antimony (20 mg/kg IV once daily) starting on Day 0.

Sequential enrollment of cohorts: * = after Day 84 of cohort 1; ** = after Day 84 of cohort 2.

**Figure 5:**
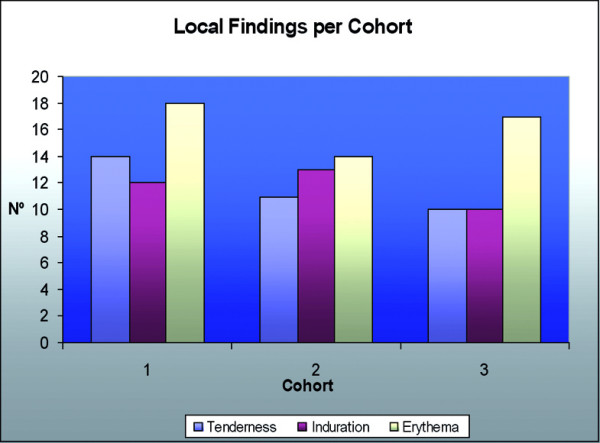
Site of injection reactions following vaccination with Leish-111f + MPL-SE.

**Figure 6:**
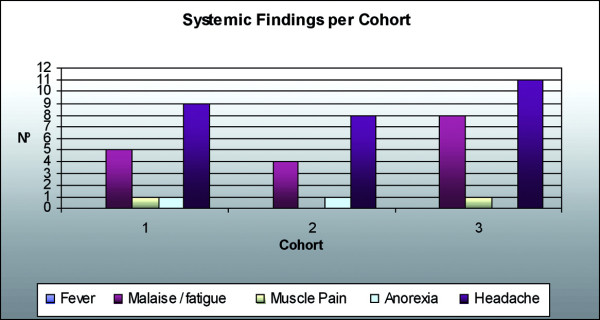
Systemic Reactogenicity following vaccination with Leish-111f + MPL-SE.

Safety assessment included observation for adverse events (clinical and laboratory) from days 0–84 and on days 168 and 336. The local and systemic reactions are shown in Figs. 5 and 6 respectively. The evolution of lesions is assessed during the 84 days, endpoint clinical response is assessed on day 84, and subsequent relapse is recorded through to day 336. Immunological responses are evaluated on days 0 (baseline), 84 and 168. Reactogenicity of the vaccine is assessed by evaluating tenderness, induration and erythema at the site of injection and systemic reactions such as fever, malaise/fatigue, generalized muscle pain, anorexia and headache.

In cohort 1 after the first injection, one patient had transaminase increase (a grade 4 AE) dose-limiting toxicity (DLT) attributed to antimony treatment and unrelated to study injection and was given no further injections. In cohort 1 at one year follow-up of final study visit, 5 patients were considered as failed and received amphotericin B (AmphB) rescue treatment; three patients were cured, and two were rescued. In cohort 2, all 16 patients received three study injections; at the 6-month follow-up no significant AE has been observed. Four patients failed and received AmphB rescue treatment, three were cured and one was under treatment. In cohort 3, 15 of 16 patients received all three study injections. One had a grade 4 AE/DLT (increased transaminases, attributed to antimony treatment and unrelated to study injection) after the first study injection and was not given further injections. At the time this presentation 15 had completed the day-84 visit. Three patients failed and had to be rescued with AmphB.

### Dose-escalating, safety and immunogenicity trial in patients with CL in Brazil

Dr Piazza presented this trial as Prof E. Nascimento (principle investigator of the Brazil trial) could not be present. The overall design of the study is similar to that above, in healthy individuals in the US, except for the lower dose range (5 μg, 10 μg and 20 μg instead of 10 μg, 20 μg, and 40 μg in healthy adults). The cohorts and injection assignments are shown in Table 2.

**Table 2:** Treatment Groups in Patients with Cutaneous Leishmaniasis (Brazil)

**Cohort**	**N**	**Treatment**	**Timing of Treatment**	
			
			**Study Injections**	**Pentavalent Antimony (Sb^5+^)**
# 1	9 3 3	5 μg Leish-111f in 25 μg MPL-SE + Sb^5+^ 25 μg MPL-SE + Sb^5+^ Placebo (saline) + Sb^5+^	Day 0, Day 28 and Day 56	SMC^1^
# 2*	9 3 3	10 μg Leish-111f in 25 μg MPL-SE + Sb^5+^ 25 μg MPL-SE + Sb^5+^ Placebo (saline) + Sb^5+^	Day 0, Day 28 and Day 56	SMC^1^
# 3**	9 3^2^ 3	20 μg Leish-111f in 25 μg MPL-SE + Sb^5+^ 25 μg MPL-SE + Sb^5+^ Placebo (saline) + Sb^5+^	Day 0, Day 28 and Day 56	SMC^1^

^1^SMC = Standard multiple cycles: 10 days of pentavalent antimony (10 mg/kg IM or IV once daily) starting on Day 0, followed by 11 days of no antimony. Cycles are repeated until cure is achieved.

^2^Only 2 patients were randomized to adjuvant in the third cohort (inability to recruit 15 patients). Sequential enrollment of cohorts: * = after Day 84 of cohort 1; ** = after Day 84 of cohort 2.

The status of this ongoing double-blind study at the time of presentation was summarized as follows:

No patient is doing worse and most patients appear to be doing better than anticipated with antimony treatment alone. The study remains blinded and immunological evaluation is in progress.

The ML trial in Peru and the CL trial in Brazil were both completed successfully in 2006. In both trials the vaccine was shown to be safe and immunogenic. Final reports and manuscripts are in preparation.

### Immune responses of healthy volunteers to Leish-111f + MPL-SE

Dr R Coler described clinical immune monitoring of phase-1 clinical trials of Leish-111f + MPL-SE (in USA, Peru and Brazil, the latter two still coded) under conditions of good clinical laboratory practices. In the US trial, 90 normal serum donor samples and 30 normal PBMC donor samples were run together with samples from volunteers in the trial. All assays were qualified. Tests included antibody by ELISA, T-cell proliferation, and IFN-γ and IL-5 measurement. Criteria for plate acceptance, and cut-off values for positive and negative responses, were established. Responses against Leish-111f and each of its components (LmSTI1, TSA, LeIF) were determined. Blood samples were collected from volunteers on days 0 (baseline), 28 (prior to first booster), 56 (prior to second booster), 84 (4 weeks post last study injection), 168 (4 months post last study injection, at the 6-month follow-up) and 336 (1-year follow-up).

From the trial on healthy volunteers in the US (see above), results can be summarized as follows:

• High IgG antibodies (dose related) to Leish-111f were seen which were generally dose related and increased with booster injections.

• All volunteers produced antibody to Leish-111f by day 56 and remained positive on day 84.

• Antibodies to the three components of Leish-111f were similarly detected (LmSTI1>TSA>LeIF), although at lower titres than the whole antigen.

• For cellular responses, unseparated PBMC were tested in a standard five-day lymphoproliferative assay with different doses of Leish-111f, the three components, phytohemagglutinin A (PHA), and several control proteins. Supernatants of cultures were assayed by ELISA for IFN-γ and IL-5.

By day 84, 100% of volunteers in the first two cohorts who had received 10 μg or 20 μg Leish-111f had a positive lymphoproliferative response. In contrast, only 75% in cohort 3 (highest vaccine dose, 40 μg Leish-111f) had positive stimulation indices on day 84, although 100% were positive on day 56 when 88% and 78% had positive stimulation index (SI) in cohorts 1 and 2 respectively. In the classical cytokine assay, 12/26 and 20/26 volunteers were positive for IFN-γ and IL-5 respectively, when cells were stimulated with Leish-111f. The same but weaker responses were seen with antigen components. The magnitude of responses were Leish-111f>LSTI1>TSA>LeIF. Higher doses of the vaccine antigen did not seem to induce higher cytokine responses. Indeed, there was a decrease in responders at the highest dose. However, IFN-γ responses increased after the first booster injections; the increase was smaller after the second booster.

To study longevity of immune response, blood samples were collected and assayed six months (day 168) and one year (day 336) after vaccination. IgG antibodies were present in 100% of volunteers by day 168, and 89%–100% by day 336 in different cohorts, indicating a long-lasting B-cell response without further exposure to antigens. On day 168, 100%, 100% and 75% of volunteers had positive proliferative indices in cohorts 1 (10 μg), 2 (20 μg) and 3 (40 μg) respectively. By day 336, the responses were 75%, 56% and 62% for the same cohorts. For IFN-γ, there were 25%, 22% and 22% responders on day 168 in cohorts 1, 2 and 3 respectively; and 50%, 11% and 25% responders on day336 in the same cohorts. For IL-5, there were 62%, 78% and 75% responders on day 168, and 62%, 44% and 88% responders on day336 in cohorts 1, 2 and 3 respectively. These data indicate that three injections of the vaccine induce humoral and cellular responses that remain positive for up to one year post vaccination in some volunteers.

**Figure 7:**
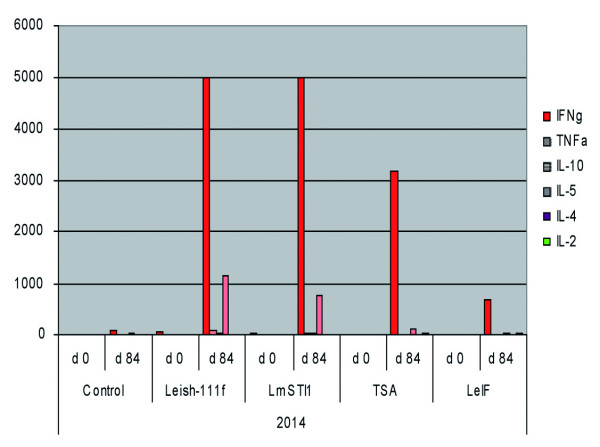
Cytometric bead array evaluation of supernatants for different cytokines.

Using the cytometric bead array system for several cytokines on the same supernatants from day-84 samples, the predominance of the IFN-γ response over IL-5 and other cytokines was demonstrated (Fig. 7). In general, there was considerable variation in the magnitude of cytokine responses amongst volunteers so, given the small sample size, these data must be regarded as trends rather than statistically significant differences.

**Figure 8:**
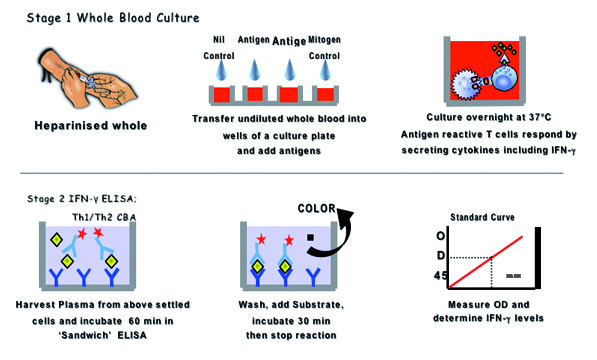
Schematic representation of whole blood culture for assays of cytokine.

Dr Coler also presented some data on antibody responses in the Peru study, though these data are coded. There were positive, albeit weak, responses to Leish-111f in some samples. For the Peru and Brazil studies, a whole blood culture technique for cytokine production was described (Fig. 8).

Dr Coler outlined her future plans using whole cell cultures and cytometic bead array (CBA) as follows:

• Assess the duration of B and T-cell responses, in particular of memory T cells upon re-exposure to leishmanial antigens.

• Measure classes and subclasses of antibodies (IgG1, IgG4, other Igs, IgE).

• Test the available supernatants of cultures for additional cytokines using the CBA method.

## Recommendations

### Treatment of PKDL

1. Based on the highly significant results of the hospital-based trial with alum-ALM + BCG in Sudan, an expanded trial under field conditions is recommended. Before the trial, availability of the vaccine, and its production under good manufacturing practices (GMP) and hence quality control, must be assured.

2. Considering the safety profile of Leish-111f + MPL-SE in healthy and active cases, the product profile documents generated by IDRI should be submitted to the Sudanese Authorities for obtaining clearance to conduct a safety-immunogenicity trial in patients with PKDL. Only one dose (three injections of 10 μg Leish-111f) should be considered and the protocol previously developed by TDR and IDRI should be updated accordingly.

3. Considering the immuno-stimulatory activities of sodium stibogluconate (SGG), the initial trials should use SSG. Subsequently, other agents (Miltefosine, Aminosidine, Ambisome) should be considered.

4. Should combination therapy be successful, the therapeutic efficacy of vaccines (alone) should be considered.

5. The ultimate goal is to prevent VL. Prophylactic trials with the vaccines should be planned if the vaccines are shown to be effective for PKDL therapy.

### Prevention of PKDL

This is one objective of using Leish-111f + MPL-SE:

1. Timing of vaccination during chemotherapy is crucial considering the immunological and haematological/biochemical status of most VL patients at the start of treatment. It is important to follow the responses (including extensive cytokine profiles) of VL patients during chemotherapy in order to decide when to introduce immunotherapy.

2. Some VL patients develop PKDL while under treatment with SSG. A close immunological analysis of these patients might reveal markers associated with high risk of PKDL development.

### Treatment of VL

Shortening the time of VL treatment, reducing the dose of medications, and preventing resistance are part of the second objective for use of Leish-111f + MPL-SE:

1. A single dose of Ambisome has been reported to cure over 90% of cases of VL in India. Addition of a vaccine to the treatment is recommended for preventing resistance to Ambisome. Time of introducing the vaccine is important (see objective 1 on prevention of PKDL). Resistance to any drug is bound to happen when single drug therapy is used.

2. It is important to prevent resistance to Miltefosine and Aminosidine, two valuable drugs that are being considered for leishmaniasis control on the Indian sub-continent. Safety trials of combination therapy should be considered with urgency. Again the time to inject the vaccine is important (see objective 1 on prevention of PKDL).

3. Once combined therapy is shown to be advantageous, and with the information gained through immunological studies of VL patients under therapy, immunotherapy alone can be considered. The ethical aspects should receive particular attention.

4. It is highly desirable to reduce the treatment period of VL in Sudan. Shorter periods of SSG (15 days instead of 30) with or without a vaccine should be studied.

## Authors' contributions

All authors read and approved the final manuscript. HG organized the meeting, presented leishmaniasis background information, lead the discussions and deliberations and reviewed the manuscript. FM presented information on leishmaniasis first generation vaccines, contributed to discussions, wrote the report of the meeting, incorporated contributors comments, prepared the final manuscript.

## Supplementary Material

Additional file 1 List of contributors. The document contains the full list and emailaddresses of all contributors. Click here for file
