# Supplementary material for: Consultation meeting on the development of therapeutic vaccines for post kala azar dermal leishmaniasis
Source: Kinetoplastid Biol Dis. 2007 Aug 17;6:7. doi: 10.1186/1475-9292-6-7 (PMC2000869; doi:10.1186/1475-9292-6-7)
Supplement: Additional file 1 — List of contributors. The document contains the full list and emailaddresses of all contributors. [file 1475-9292-6-7-S1.doc]

**Participants and contributors:**

**Jorge Alvar,** NTD/CDS, [alvarj@who.int](mailto:alvarj@who.int);

**Jill Ashman,** Infectious Disease Research Institute (IDRI), USA, [jashman@idri.org](mailto:jashman@idri.org); **Roberto Badaro,** University of California San Diego, USA, [rbadaro@ucsd.edu](mailto:rbadaro@ucsd.edu); **Sylvie Bertholet,** Infectious Disease, Research Institute (IDRI), USA, [bertholets@idri.org](mailto:bertholets@idri.org);

**Rhea Coler,** Infectious Disease Research Institute (IDRI), U SA, [Coler@idri.org](mailto:Coler@idri.org); **Ahmed Mohammed El Hassan,** University of Khartoum, Sudan, [ahmedelhassan@iend.org](mailto:ahmedelhassan@iend.org);

**Hashim Warsama Ghalib,** RCS/TDR/WHO, [ghalibh@who.int](mailto:ghalibh@who.int);

**Eltahir Awad Gasim Khalil,** University of Khartoum, Sudan. [eltahirk@iend.org](mailto:eltahirk@iend.org); **Marie-Paule Kieny,** Director, IVR/WHO, [kienym@who.int](mailto:kienym@who.int);

**Juntra Laothavorn,** Clinical Coordinator, TDR/WHO, [karbwangj@who.int](mailto:karbwangj@who.int);

**Janis Karlin Lazdins-Helds,** Acting Coordinator PDE/TDR/WHO, [lazdinsj@who.int](mailto:lazdinsj@who.int);

**Alejandro Llanos-Cuentas,** Universidad Peruana Cayetano Heredia, Perú, [allanos@conhu.org.pe](mailto:allanos@conhu.org.pe);

**Farrokh Modabber,** Tehran University of Medical Sciences, Iran (Islamic Republic), [modabberf@yahoo.com](mailto:modabberf@yahoo.com);

**Ahmed Mudawi Musa,** University of Khartoum, Sudan, [amusa@iend.org](mailto:amusa@iend.org);

**Evaldo Nascimento,** Brazil, [evaldo@icb.ufmg.br](mailto:evaldo@icb.ufmg.br);

**Sassan Noazin,** CDS/EPR/ARO/ERI, [noazins@who.int](mailto:noazins@who.int);

**Franco Piazza,** Infectious Disease Research Institute (IDRI), USA, [fpiazza@idri.org](mailto:fpiazza@idri.org);

**Piero Luigi Olliaro,** PDE/TDR, [olliarop@who.int](mailto:olliarop@who.int);

**Steven G. Reed,** Infectious Disease Research Institute, USA, [reeds@idri.org](mailto:reeds@idri.org);

**Robert George Ridley,** Director, TDR/WHO, [ridleyr@who.int](mailto:ridleyr@who.int);

**Shyam Sundar,** India, [shyam_vns@sify.com](mailto:shyam_vns@sify.com);

**Marian Ulrich,** Instituto de Biomedicina, Venezuela, [mulric@telcel.net.ve](mailto:mulric@telcel.net.ve);

**Edward Zijlstra,** College of Medicine, Malawi, [eezijlstra@malawi.net](mailto:eezijlstra@malawi.net)
